# Ontogenetic and phylogenetic simplification during white stripe evolution in clownfishes

**DOI:** 10.1186/s12915-018-0559-7

**Published:** 2018-09-05

**Authors:** Pauline Salis, Natacha Roux, Olivier Soulat, David Lecchini, Vincent Laudet, Bruno Frédérich

**Affiliations:** 1 Observatoire Océanologique de Banyuls-sur-Mer, UMR CNRS 7232 BIOM, Sorbonne Université Paris, 1, Avenue Pierre Fabre, 66650 Banyuls-sur-Mer, France; 2 Aquarium de Canet-en-Roussillon, 2 Boulevard de la Jetée, 66140 Canet-en-Roussillon, France; 3 EPHE-UPVD-CNRS, USR3278 CRIOBE, PSL Research University, BP 1013, 98729 Papetoai, Moorea French Polynesia; 4 Laboratory of Functional and Evolutionary Morphology, FOCUS, University of Liège, Liège, Belgium

**Keywords:** Ontogeny, Diversification, Pomacentridae, Color evolution, Adaptive coloration

## Abstract

**Background:**

Biologists have long been fascinated by the striking diversity of complex color patterns in tropical reef fishes. However, the origins and evolution of this diversity are still poorly understood. Disentangling the evolution of simple color patterns offers the opportunity to dissect both ultimate and proximate causes underlying color diversity.

**Results:**

Here, we study clownfishes, a tribe of 30 species within the Pomacentridae that displays a relatively simple color pattern made of zero to three vertical white stripes on a dark body background. Mapping the number of white stripes on the evolutionary tree of clownfishes reveals that their color pattern diversification results from successive caudal to rostral losses of stripes. Moreover, we demonstrate that stripes always appear with a rostral to caudal stereotyped sequence during larval to juvenile transition. Drug treatments (TAE 684) during this period leads to a dose-dependent loss of stripes, demonstrating that white stripes are made of iridophores and that these cells initiate the stripe formation. Surprisingly, juveniles of several species (e.g., *Amphiprion frenatus*) have supplementary stripes when compared to their respective adults. These stripes disappear caudo-rostrally during the juvenile phase leading to the definitive color pattern. Remarkably, the reduction of stripe number over ontogeny matches the sequences of stripe losses during evolution, showing that color pattern diversification among clownfish lineages results from changes in developmental processes. Finally, we reveal that the diversity of striped patterns plays a key role for species recognition.

**Conclusions:**

Overall, our findings illustrate how developmental, ecological, and social processes have shaped the diversification of color patterns during the radiation of an emblematic coral reef fish lineage.

**Electronic supplementary material:**

The online version of this article (10.1186/s12915-018-0559-7) contains supplementary material, which is available to authorized users.

## Background

Understanding the diversification of phenotypes requires to integrate developmental and evolutionary analysis in an ecological context [1]. Having a well-defined phylogenetic context is essential to recognize the pattern of trait evolution as well as to detect events of parallel or convergent evolution. In addition, studying how phenotypic traits differ across natural environments as well as their adaptive value allows to reveal the factors shaping the emergence of diversity. Lastly, the study of trait development helps to identify the molecular mechanisms behind phenotypic diversification as well as constraints that bias their evolutionary trajectories.

Pigmentation, in particular color patterns, provides an incredible number of cases that allow the exploration of the interplay between ecology, evolution, and development that are at the basis of trait diversification [2–6]. Among vertebrates, coral reef fishes provide classical examples of complex color patterns exhibiting a huge variety, and therefore, they offer a unique opportunity to better understand, in an integrated manner, the origin of those traits [7]. Most of coral reef fish species display spots, stripes, repeated lines, eyespots, grids, etc. This diversity in color patterns serves for species recognition [8, 9], camouflage [10, 11], mimicry [12], and/or warning [13]. For example, the eyespots of the damselfish *Pomacentrus amboinensis* have been suggested to serve as a subordinate signal directed to dominant males [14]. To date, work on coral reef fishes has mainly been focused on the link between color patterns, ecology, and behavior, that is, the ultimate role of these patterns [15]. However, the underlying development controlling these patterns and their evolution, that is, their proximal mechanism, is still largely unknown [15, 16].

It is now well known that phenotypic diversification between lineages may be achieved by changes in developmental processes [1, 17]. There are a number of possible developmental mechanisms that explain how specific changes in signaling pathways can induce phenotypic changes between lineages, and a main goal of Evo/Devo is to better understand these processes. Within this framework, various studies devoted to the pigmentation of zebrafish allowed to pinpoint changes in developmental mechanisms leading to color variation among related fish species [18–20]. However, the incredibly diverse color patterns of coral reef fishes have never been explored with such an Evo/Devo perspective. Despite this, there are some evidences that developmental processes may indeed sustain the diversification of color patterns in some species. For example, the polymorphic damselfish *Chrysiptera leucopoma* may retain its juvenile color (a bright yellow body with a dorsal blue line) or shift to the adult phenotype (a dark brown body) depending on habitat type and/or population densities [21]. However, in this example, no study of the underlying developmental mechanisms has been performed.

Clownfishes (*Amphiprion* and the monotypic *Premnas*) are iconic coral reef fishes [22]. This tribe (Amphiprionini; [23]) within Pomacentridae is composed of 30 species that display a relatively simple color pattern made of zero to three white vertical stripes that are well visible on a yellow to red, brown, or even black body background [22]. Their life cycle includes a relatively short dispersive planktonic larval phase in the open ocean [24], followed by the settlement of juveniles into sea anemones where they live in a social group composed of a dominant breeding pair and a varying number of sexually immature subalterns [22]. The functional role of striped patterns in clownfishes is still unknown but could be associated with predator defense, foraging mode, macro-habitat type, species recognition, etc. as observed in various teleosts [15, 25]. The relatively simple color pattern of *Amphiprion* offers a good opportunity to better delineate the patterns and processes allowing the diversification of such ornamental diversity. The clownfish evolutionary radiation has recently received much attention, providing a suitable phylogenetic framework for testing new evolutionary hypotheses on the rise of color diversity in coral reef fishes [26].

In this study, we focus on the vertical white stripes present in most species of *Amphiprion*. We first map their occurrence and pattern on the clownfish evolutionary tree and reconstruct the ancestral state in terms of white stripe presence/absence. Our results provide evidences that the diversification of clownfish color pattern results from successive caudal to rostral losses of stripes during evolution. Using specific drugs (e.g., TAE 684: an inhibitor of tyrosine kinase receptors expressed in zebrafish iridophores), we reveal that the white stripes are formed by iridophores and are essential for the patterning of the neighboring black stripes. Then, by an ontogenetic approach, we show that either the juvenile has the same number of stripes than adults or the juvenile has supplementary stripes that disappear caudo-rostrally later on. The reduction of stripe number over ontogeny totally matches the sequences of stripe losses across evolution, demonstrating that the diversification in color pattern among clownfish lineages results from changes in developmental processes. Finally, we determine the links between the number of stripes and other external morphological traits, and we provide some evidence that the various striped patterns have evolved for species recognition. This approach allows us to consider the relationships among striped patterns, fish morphology, and their ecology to suggest that both developmental and ecological processes have shaped the diversity of color patterns in clownfishes.

## Results

### Successive caudo-rostral loss of stripes during evolution

**Fig. 1:**
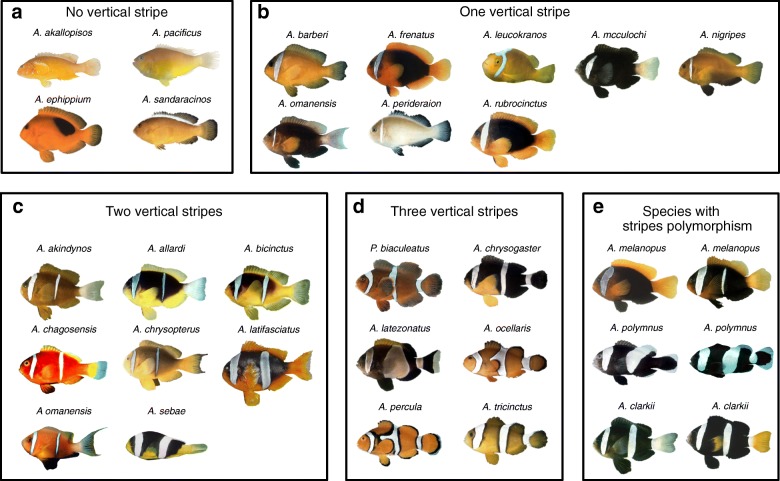
Adult color patterns of clownfish species. Pictures of adult clownfishes classified depending on their color patterns. **a** No vertical stripe, **b** one vertical stripe on the head, **c** two vertical stripes (one on the head, the other on the body), **d** three vertical stripes (one on the head, one on the body trunk, and the last one on the peduncle), **e** fishes having stripes polymorphism

Clownfishes can be classified into four categories according to their striped pattern at the adult stage: species without vertical stripe (group A) or species having one white vertical stripe (on the head—group B), two vertical stripes (on the head and the trunk—group C), or three vertical stripes (head, trunk, and caudal peduncle—group D) (Fig. 1 and Additional file 1: Table S1). Interestingly, there is no species with a single stripe on the trunk or on the peduncle (Fig. 1). White stripe on the trunk is always associated with a head stripe. The white stripe on the peduncle is always preceded by stripes on the head and the trunk.

**Fig. 2:**
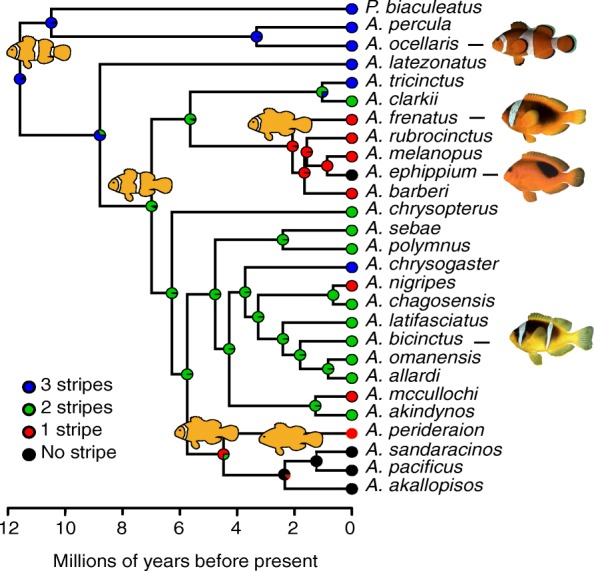
Successive caudo-rostral loss of stripes during evolution. Phylogenetic tree of clownfishes from Litsios et al. 2014 [26] with a summary map of white stripe number histories generated through stochastic character mapping. This trait mapping shows that the diversification of white striped pattern is a history of loss from an ancestral clownfish having three stripes and that these losses occurred in a progressive and sequential fashion from caudal to rostral. Circles at the tips of the tree indicate each species striped pattern and circles at every internal nodes give probabilities of ancestral striped pattern

To understand the evolution of color pattern in clownfishes, we performed a stochastic mapping of striped patterns on the most complete time-calibrated phylogeny of Amphiprionini [27]. The analysis highly suggests that the common ancestor of extended clownfishes exhibited three vertical white stripes (90–100% of posterior probabilities; Fig. 2), independent from the color pattern polymorphism of some species (Additional file 2: Figure S1). The state reconstruction for every internal node of the phylogeny illustrates successive losses of vertical stripes from the caudal to the rostral region (Fig. 2). The stochastic mapping, which was performed with a model assuming that all transition rates are free to vary, shows that some transitions between stripe morphs never occur. The evolutionary loss or gain of two white stripes appears very unlikely but the gain of one stripe, i.e., reversion, is possible (e.g., *Amphiprion chrysogaster*—Fig. 2 and Additional file 3: Table S2). Thus, we tested these hypotheses by comparing the fit of four evolutionary models varying in their matrix of transition rates between stripe patterns using the Multiple State Speciation Extinction (MuSSE) method [28, 29]. In these four models, rates of speciation and extinction were constrained to be equal among stripe morphs in order to reduce the number of model parameters. The best-fitting models according to the Akaike Information Criterion are the most constrained models iii and iv (Table 1 and Additional file 4: Table S3). Both models assume that the loss or the acquisition of two stripes is not allowed. Moreover, in the great majority of the scenarios tested (Additional file 4: Table S3), model fitting highly suggests that the transition rates among stripe morphs are symmetric, i.e., the rate shift from stripe morph A to B is equal to the rate shift from stripe morph B to A. The estimation of transition rates from the best-supported model (model iv) suggests that the appearance/disappearance of the third stripe on the caudal peduncle occurred more slowly (mean ± SD = 0.052 ± 0.001) than the one of the second stripe in the trunk (0.103 ± 0.001) or the first in the head (0.123 ± 0.001).

**Table 1:** Model fitting of the four striped pattern evolutionary models

	Transition matrix	AIC	∆AIC	wtAIC
Model iv	Model ii + model iii	191.74	0.00	0.88
Model iii	Loss or gain of two stripes are forced to be 0	196.58	4.84	0.08
Model ii	Symmetric rates of transition among states	197.74	6.00	0.04
Model i	Free	205.58	16.84	0.00

Models are ranked from best to worst, according to AIC scores and Akaike weights (wtAIC). ∆AIC scores indicate the difference between the candidate model and the best-fitting model. According to [58], a ΔAICc value of 4 or more was taken as an indication of support for one model over the other following. Matrices of transition rates (q) among stripe morphs are described for the four evolutionary models. Speciation and extinction rates are assumed to be equal among morphs

This evolutionary analysis therefore highlights that the diversification of white stripe pattern in clownfishes is a history of loss from an ancestral lineage having three stripes and that these losses always occurred in a progressive and sequential fashion from caudal to rostral regions. For example, in all two striped species, the peduncle stripe has been lost and the head and the trunk stripes are retained. All one-stripe species retained the head stripe and have lost the peduncle and trunk stripes.

### Ontogeny of stripe formation reveals a rostro-caudal stereotyped pattern

The fact that stripes always appear at the same location in clownfishes and that the losses of stripes during evolution occurred in a sequential manner from caudal to rostral suggests that this loss may be constrained by a developmental mechanism. We thus tested whether a variation in the number of stripes could occur during clownfish ontogeny by studying the development of *Amphiprion ocellaris* and *Amphiprion frenatus* that display three stripes (i.e., similar to the ancestral state) or a single head stripe at the adult stage, respectively.

**Fig. 3:**
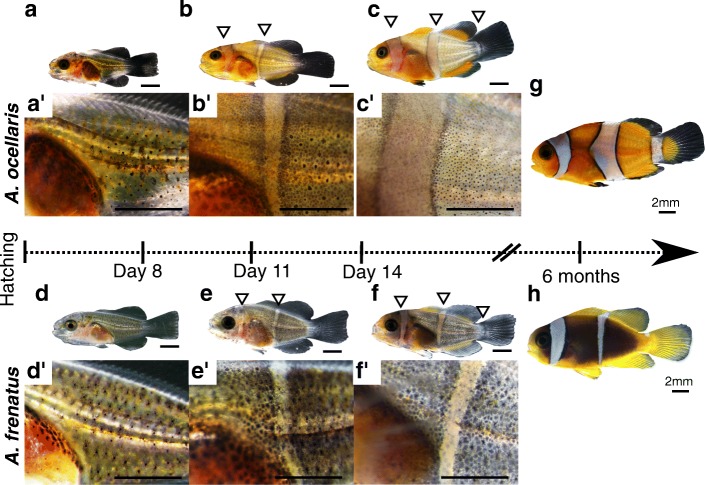
Ontogeny of stripe formation reveals a rostro-caudal stereotyped pattern. *A*. *ocellaris* (a–c′ and g) and *A*. *frenatus* (d–f′ and h) color pattern ontogenesis at 8 dph (a-a′, d-d′, *A*. *ocellaris*: *n* = 10; *A*. *frenatus*: *n* = 3), 11 dph (b-b′, e-e′, *A. ocellaris*: *n* = 10; *A*. *frenatus*: n = 3), 14 dph (c-c′, f-f′, *A. ocellaris*: *n* = 10; *A. frenatus*: *n* = 3) and 6 months post hatching (g and h, *n* = 5). Higher magnification of the medial white stripe ontogenesis (a′, b′, c′, d′, e′, f′). Note that the white stripes appear in the same rostral to caudal sequence in both species. Scale bars correspond to 1 mm

At 8 days post hatching (dph), larvae of both species do not harbor any vertical stripe (Fig. 3a-a′ and d-d′). At 10–11 dph, *A*. *ocellaris* larvae acquire the head and trunk stripes simultaneously (Fig. 3b). Surprisingly, the same is true for *A*. *frenatus* (Fig. 3e-e′). In *A*. *ocellaris*, the third stripe on the caudal peduncle is formed at 14 dph (Fig. 3c). Strikingly, we also observed the development of a third stripe on the caudal peduncle of some *A*. *frenatus* at the same larval period, as in *A*. *ocellaris* (Fig. 3f). In our husbandry conditions, larvae of *A*. *frenatus* reach the juvenile stage with either two or three stripes (Fig. 3h): the anterior one on the head, the medial one on the trunk, and the posterior one on the caudal peduncle whereas *A*. *ocellaris* reach the juvenile stage with three stripes (Fig. 3g). The loss of the trunk stripe will occur after several months and is therefore a prominent feature of juvenile *A*. *frenatus*.

Our data provide strong evidence that there are two distinct and inverse phenomena: (i) an evolutionary pattern of stripe loss, with a caudo-rostral progression that is observed in adult fish, and (ii) a developmental pattern of rostro to caudal stripe gain during the larval to juvenile transition exemplified in *A*. *ocellaris* and *A*. *frenatus* followed by a sequential loss of caudo-rostral during juvenile stage in some species such as *A*. *frenatus*. Taken together, these results emphasize the fact that clownfish color pattern evolution is constrained by developmental processes that may also explain why there is no species with a single stripe on the tail or trunk.

**Fig. 4:**
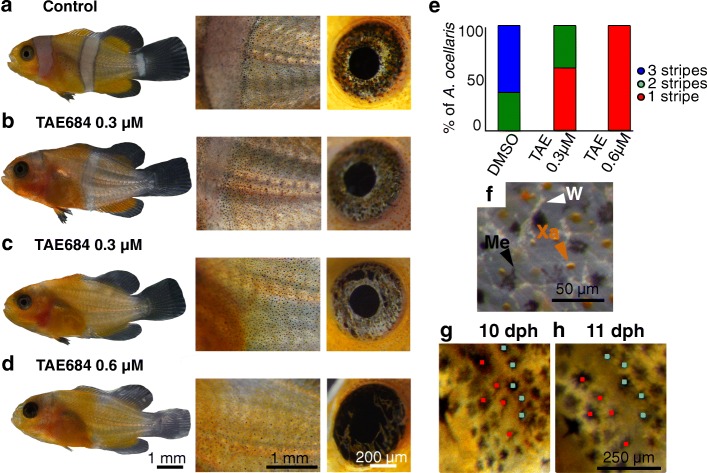
Cellular mechanism of color pattern ontogenesis in *A*. *ocellaris*. **a**–**d** Dose-dependent modifications of color pattern (left and middle panel) and iridescence of the eye (right panel) after 13 days of TAE684 drug treatment of *A*. *ocellaris* at 18 dph at 0.6 μM (**d**) and 0.3 μM (**b**, **c**) compared to DMSO (control, **a**). **e** Cumulative histogram of fishes having fully stripes: one stripe (head—red), two stripes (head and trunk—green), or three stripes (blue) in control (*n* = 6), TAE 0.3 μM (*n* = 16) and TAE 0.6 μM (*n* = 3). **f** Stereomicroscope pictures showing the three types of chromatophores within the trunk of juvenile *A*. *ocellaris*. (**g**, **h**, *n* = 4). Live imaging pictures of the same *A*. *ocellaris* individual at 10 dph (**g**) and 11 dph (**h**) show that during medial white stripe formation, the distance increases between melanophores underlined with red dots and melanophores underlined with blue dots

In order to better understand the white stripe ontogenesis, we examined the cellular process by which the stripes are formed. In teleost fishes, different types of pigment cells (or chromatophores) are described according to their ultrastructure and their pigment type [30]. Xanthophores (orange cells), iridophores (white and iridescent cells), leucophores (white), and melanophores (black cells) are the four main characterized cells. In *A*. *ocellaris*, we observed at least three types of cells: xanthophores, melanophores, and white cells (Fig. 4f). From juvenile to adult stage, orange skin is composed of xanthophores and round melanophores. The white stripe comprises stellar melanophores and white cells whereas the black stripes are formed of a dense number of melanophores (Fig. 4a). To determine if leucophores or iridophores are involved in white stripe formation in *A*. *ocellaris*, we tested whether the drug TAE684 (TAE), known as an inhibitor of leukocyte tyrosine kinase (Ltk) and anaplastic lymphoma kinase (Alk) and leading to a decrease of iridophore number in zebrafish [31], could disrupt white stripe formation. Treatments with TAE at 0.6 μM or 0.3 μM during the metamorphosis of *A*. *ocellaris* larvae (i.e., from 5 until 18 dph) induced a dose-response effect into the formation of white stripes (Fig. 4a–d). While the control develops the head, the trunk, and the peduncle white stripes (Fig. 4a), fish treated with 0.6 μM TAE develop a complete transparent head stripe whereas the body stripe is uncompleted. This suggests that there is a decrease in the number of iridophores (Fig. 4d, e). Fish treated with 0.3 μM show an intermediate phenotype with 50% of the fish having the same phenotype as fish treated with 0.6 μM (Fig. 4c, e) and 50% having full transparent head and trunk vertical stripes (Fig. 4b, e). Similarly, a dose-response effect on the iridescence of the eye was observed (Fig. 4 right panel a–d). Indeed, whereas the eyes of fish control and fish treated at 0.3 μM TAE are iridescent (Fig. 4a–c), those of fish treated at 0.6 μM TAE are blackish (Fig. 4d). Together, these results demonstrate that white cells correspond to iridophores but also that Ltk and/or Alk is required for the formation of iridophores and color pattern during metamorphosis of *A*. *ocellaris*.

TAE treatments during the metamorphosis of *A*. *ocellaris* larvae allow the understanding of how iridophores contribute to color pattern development. White vertical stripes are surrounded by a thin stripe of melanophores over an orange body. Interestingly, we observe that when no white vertical stripe is formed over the body (Fig. 4d), melanophores are dispersed with xanthophores over the flank and they do not form any stripe. This is interesting to link to this observation that, during formation of the white stripe in control fish, iridophores initially appear at the future stripe location and push black melanophores at the periphery to form stripe pattern (Fig. 4g, h). This suggests that melanophores are expelled from the white stripe in normal condition to form the black stripes. In addition, we observed that xanthophores are not localized at their proper location after exposure with TAE. These data not only reveal that the cellular substrate underlying the white color is likely based on iridophore cells but also that they shed light on the fact that specific interactions between different chromatophore types play an important role in the stripe formation in *A*. *ocellaris*.

### Stripe loss during ontogeny occurs multiple times in *Amphiprion*

**Fig. 5:**
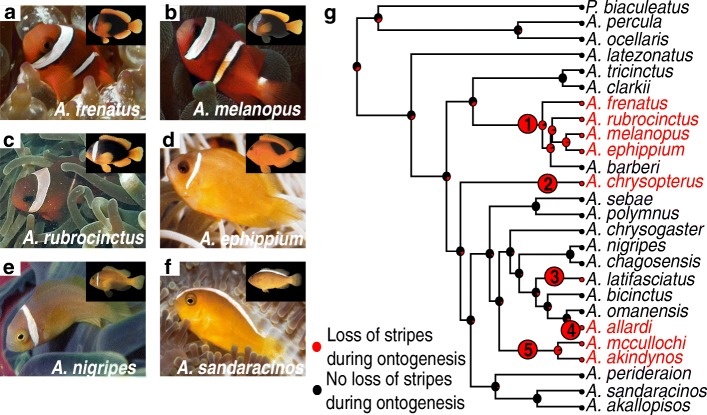
Stripe loss during ontogeny occurs multiple times in *Amphiprion.* **a**–**f** Pictures of juveniles (big picture) and adult (small picture-top right) of *A*. *frenatus* (**a**), *A*. *melanopus* (**b**), *A*. *rubrocinctus* (**c**), *A. ephippium* (**d**), *A*. *nigripes* (**e**), and *A*. *sandaracinos* (**f**). *A. frenatus* (**a**), *A*. *melanopus* (**b**), *A*. *rubrocinctus* (**c**), and *A. ephippium* (**d**) show that juveniles have extra stripes compared to its respective adult whereas the number of vertical stripes does not vary over ontogeny in *A*. *nigripes* (**e**) and *A*. *sandaracinos* (**f**). Pictures of juveniles were nicely provided by GR Allen. **g** Maximum clade credibility phylogeny of clownfishes [27] with a summary map of striped pattern ontogenesis generated through stochastic character mapping. It reveals a minimum of five major transitions to an ontogenetic pattern made of white stripe loss, occurring (1) in the *A*. *frenatus* clade, (5) in the clade grouping *A*. *mccullochi* and *A*. *akindynos*, and in three individual species (2) *A*. *chrysopterus*, (3) *A*. *latifasciatus*, and (4) *A*. *allardi* (number in red circles)

The observation that *A*. *frenatus* juveniles have more stripes than adults prompted us to further document the evolution of ontogenetic trajectories of color pattern in clownfishes. For this, we compiled ontogenetic information on 26 *Amphiprion* species with a multi-tiered approach utilizing the primary literature, online databases, and field observations made by various experts (Additional file 1: Table S1). We observed that a minimum of nine species show extra stripes during the juvenile phase when compared to adult. This is illustrated on Fig. 5 for four species: *A*. *frenatus*, *A*. *melanopus*, *A*. *rubrocinctus*, and *A*. *ephippium.* This contrasts with other cases (e.g., *A*. *nigripes* or *A*. *sandaracinos*; Fig. 5e, f) for which the number of stripes is invariant over ontogeny. We therefore categorized the species showing a loss of stripes during ontogeny (group 1) and the species without a loss of stripes during their development (group 2) (Additional file 1: Table S1), and we studied the evolution of these two trajectories during the evolution of clownfishes (Fig. 5g). Ancestral state reconstruction supports (64% of posterior probabilities) that the last common ancestor of extended clownfishes did not lose white stripes during ontogeny. Moreover, stochastic mapping reveals a minimum of five major transitions in the occurrence of white stripe loss during ontogeny: (i) one in the *frenatus* clade, (ii) one in *A. chrysopterus*, (iii) one in *A*. *latifasciatus*, (iv) one in *A*. *allardi*, and (v) one in the clade grouping *A*. *mccullochi* and *A*. *akindynos* (Fig. 5g, red circles). This reveals a convergence in the process of loss of stripes during ontogeny, probably triggered by selective factors (ecological, behavioral, etc.), and/or shared molecular and cellular mechanisms.

### Links between striped patterns, ecology, and external morphology

Striped patterns are adaptive and related to ecological and behavioral differences among cichlid species [25]. In butterflyfishes, striped body patterns showed correlated evolution with a number of ecological factors including habitat and sociality [15]. Clownfishes vary in their ecology [22], and one of the most striking variations among species is the diversity of sea anemone hosts. Some clownfishes are specialists, living with only one sea anemone species, while some others are generalists, capable of living in association with several host species [32]. To establish if striped patterns of clownfishes are related to this ecological difference, we first tested the simple prediction that the number of white stripes is related to the number of possible sea anemone hosts using phylogenetic generalized least-squares (PGLS) regressions. However, the number of white stripes is unrelated to the number of hosts in which they exhibit mutualistic interactions (*F* = 0.13, *P* = 0.72; Additional file 5: Table S4).

**Fig. 6:**
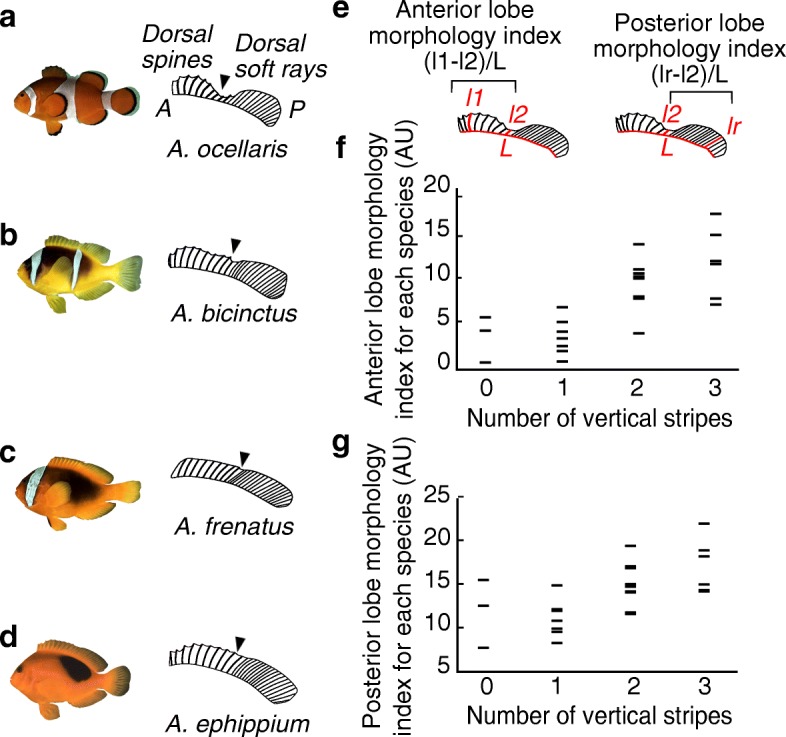
Morphological trait analysis reveals a link between striped pattern and shape of the dorsal fin. **a**–**d** Pictures of *A*. *ocellaris* (**a**), *A*. *bicinctus* (**b**), *A*. *frenatus* (**c**), and *A*. *ephippium* (**d**) and cartoons illustrating their dorsal fin shape (A anterior, P posterior). There is an indentation at the middle of the dorsal fin (black arrowhead) with the anterior spiny rays longer than the most posterior one in clownfishes having two or three stripes. **e** Methods for quantification of anterior lobe and posterior lobe morphology index (l1 length of the third dorsal spine, l2 length of the most posterior spine, lr length of the longest soft ray, L length of the dorsal fin was used for normalization). Anterior and posterior lobes morphology indexes correspond to (l1-l2)/L and (lr-l2)/L, respectively. **f**, **g** Scatterplots showing the relationship between the numbers of vertical white stripes (*x*-axis) and lobe morphologies index of the dorsal fin (*y*-axis). Each point corresponds to one clownfish species

Clownfishes also vary in their external morphology (Fig. 6), and it was suggested that this morphological disparity is related to variation in both host type and habitat partitioning [33]. At a first glance, the shape of the dorsal fin varies among clownfish species according to their white stripe patterns (Fig. 6). Indeed, an indentation at the middle of the dorsal fin in clownfish species having two and three stripes is visible (Fig. 6a, b, e–g) whereas this is less obvious in species having one and zero stripes (Fig. 6c, e–g and Additional file 6: Figure S2). Thus, we focus on fish body form, knowing that the number of color stripes might be size-dependent [34] and fin morphologies since these traits are usually linked to adaptation towards different macro-habitats (e.g., [35, 36]). We quantified body size, body elongation, and dorsal fin morphology of a minimum of 22 clownfish species and we tested whether stripe patterns are correlated with these morphological traits. Phylogenetic generalized least squares (PGLS) reveal that the evolution of the number of white stripes is unrelated to body size (*F* = 0.03, *P* = 0.87) and body elongation (*F* = 0.64, *P* = 0.43; Additional file 5: Table S4). On the other hand, PGLS analysis shows a strong correlation between the anterior lobe morphology of the dorsal fin and the number of white stripes (*F* = 14.53, *P* <  0.001) whereas the co-evolution of posterior lobe morphology and the number of white stripes is weaker (*P* = 0.05; Additional file 5: Table S4).

These analyses highlight that if there are few links between the striped pattern and clownfish body morphology, there is one between the striped pattern and dorsal fin shape. This link suggests that both traits depend on linked developmental and/or selective processes.

### Are striped patterns used for species recognition?

“Species recognition” refers to the behavior whereby individuals identify and keep track of conspecifics for group coherence or identify a suitable sexual partner [37]. Accordingly, we hypothesized that the striped patterns of clownfishes could function for “species recognition,” discouraging association of non-conspecifics and/or encouraging association of conspecifics. Application of this hypothesis predicts that sympatric species should have distinctly different striped patterns. To test this hypothesis, we counted the number of identical pairs within eight communities of sympatric clownfishes [38] and we investigated whether similarity of striped patterns among sympatric species was less than expected if communities were composed of a random set of species. Relative to the four striped patterns, the diversity of clownfish communities is consistent with this species recognition hypothesis (Table 2). Indeed, except for The Keppels (Great Barrier Reef, Australia) and Komodo, the number of identical pairs was minimal within each natural community (Table 2). Moreover, the randomization test showed that this result is unexpected in the great majority of locations given the distribution of striped patterns among species. This test only failed for Keppels and Komodo communities, which house the highest number of polymorphic species (*A*. *clarkii* and *A*. *melanopus*). Overall, these data provide evidence that the distribution of striped patterns is not random in clownfish communities, suggesting that stripes help for species recognition.

**Table 2:** Diversity of clownfish communities is consistent with the species recognition hypothesis

Location	Number of species	Maximum of identical pairs	Randomization			*P* value
			Proportion less	Proportion equal	Proportion greater	
Marshall Island	4	1	0.1393	0.6402	0.2205	< 0.0001
Japan	3	0	0.1845	0.5715	0.2440	< 0.0001
Lombok	3	0	0.1850	0.5676	0.2474	< 0.0001
Christmas Island	3	0	0	0.4472	0.5528	< 0.0001
Bali	3	0	0	0.4568	0.5432	< 0.0001
Komodo	5	3	0.5958	0.3068	0.0974	1.00
Keppels (GBR)	3	3	0.5131	0.2974	0.1895	1.00
Hoga	6	3	0.1807	0.4369	0.3824	< 0.0001

Number of pairs of identical stripe patterns in the eight clownfish communities, compared to communities assembled randomly from all clownfish species living in the western and central Pacific Provinces (i.e., a total of 10 species). The maximum number of identical pairs within each natural community was calculated by including the possibility of color polymorphism in some species

## Discussion

Our study reveals an unexpected link between the variation in the number of stripes occurring during ontogeny and the same type of variation occurring during evolution of clownfishes. Strikingly, the sequence of events observed during evolution mirror the one seen during ontogeny. An identical sequence of white stripe loss than the one observed during evolution was observed during late juvenile stages. These observations strongly suggest that the diversification of stripe patterns observed during clownfish radiation is the product of modifications of an ancestral stereotyped ontogenetic trajectory. From a mechanistic point of view, such a successive gain of stripes from the head to the caudal region during ontogeny support the hypothesis that loss of stripes during evolution is likely constrained by ontogenesis.

The sequential appearance of white stripes from the anterior to the posterior region during the development of two distantly related species, *A*. *ocellaris* and *A*. *frenatus*, is remarkable. This highly suggests a conserved mechanism of color pattern ontogeny across clownfishes. Here, we provide a first analysis into the mechanistic underpinnings of the patterns seen. In a first part, using an inhibitor of two receptor tyrosine kinases (Ltk and Alk) that are instrumental in iridophore formations in zebrafish, we show that the white coloration of stripes is produced by iridophores and not leucophores. After TAE treatments, we show that stripes are sometimes absent, incomplete, and/or less white. Disrupting the white stripe formation reveals that the presence of iridophores is instrumental for the distribution of the two other chromatophore types. In TAE-treated fish, xanthophores and melanophores are scattered on the flank and not properly organized as in wild type. This suggests that, as in zebrafish [39], cell-cell interactions are critically important for pattern generation in clownfish. Additionally, our results suggest that clownfish color pattern is not formed by a reaction/diffusion mechanism based on Turing model [40] as in zebrafish. Up to now, our knowledge of stripe formation in teleost fishes derived from studies achieved in zebrafish, the most widely used fish model species (reviewed in [41]). However, it is likely that the pattern of stripe formation in zebrafish and clownfishes are controlled by different mechanisms. In zebrafish, the color adult pattern composed of periodical horizontal blue and orange stripes is effectively formed by a reaction/diffusion mechanism which predicts the periodic pattern [40]. This model is able to regulate the width of the stripes and it explains that, during growth, new stripes insert between the preexisting ones to keep stripe width [42]. Such Turing-like model was confirmed in long-fin zebrafish mutant which continues to form perfectly new stripes as the fins grow [43] and during normal growth of *Pomacanthus imperator* [44]. The case of clownfishes is totally different since the number of stripes is fixed and independent of fish body size. Moreover, in clownfishes, new stripes do not form when the distance between two previous ones increases but following an ordered anterior-to-posterior pattern. In addition, the disappearance of stripes during growth that we observed in some clownfish species (e.g., *A*. *frenatus* and *A*. *chrysopterus*) does not fit with Turing predictions and thus allows a clear refutation of the “Turing pattern hypothesis.” This suggests that, in clownfishes, when and where the stripes must be formed are controlled by specific patterning mechanisms that remain to be analyzed.

Various clownfish species exhibit extra stripes during the juvenile phase when compared to the adult stage, and this convergent loss of stripes occurred at least five times across the evolution of clownfishes (Fig. 5). Such a convergence may be explained by shared molecular and cellular mechanisms triggering signals of stripe loss during ontogeny and/or by shared responses to selective factors (ecological, behavioral, etc.). In terms of proximal mechanisms, this loss of white stripes likely does not involve a modification of an ancestral pre-pattern but could result from different mechanisms. In zebrafish, it is known that pigment cells must be continuously formed to maintain the pattern of bands [45, 46]. The loss of white stripe observed in some clownfish species during late juvenile life may be caused by a spreading of white iridophores over the body, an extensive apoptosis of these cells, and/or a dedifferentiation of the chromatophores resulting in an absence of pigment cell synthesis.

The correlated evolution between the numbers of vertical white stripes and dorsal fin shape suggests that both phenotypic traits may depend on the same or linked developmental processes. An ecomorphological interpretation of these results would be that patterns of stripes are somehow linked to differences in macro-habitats, but this hypothesis certainly needs further analyses. On the other hand, the fact that the white stripe is present mostly in species with an indentation in the dorsal fin may suggest that it may be linked to a disruptive effect [47], the band and indentation both helping to hide the fish silhouette. This may be part of a general strategy of poor swimmer fish to reinforce their disruptive coloration to avoid being tackled by predators. However, these observations urge for additional studies in order to disentangle the relationship between the color patterns and the ecology of clownfish species.

Beyond ecological adaptation, we provide evidence that the striped patterns play a role for species recognition in clownfishes. Indeed, the number of species pairs showing the same striped pattern is exceptionally low in natural communities compared with random ones. Non-significant results from Keppels and Komodo (Table 2) are probably due to stripe polymorphism of species living in these locations, a character which was included by default in our analyses. We hypothesize that this polymorphism of stripe numbers is likely driven by the function of species recognition but this needs to be tested. The diversity of white stripe patterns may result from social selection, where signals encourage association of conspecifics [48]. Indeed, social selection on visual signals can be very strong in clownfishes, which live in social groups based on a size dominance hierarchy where agonistic interactions are numerous [49–51]. We expect that a variation in the number of stripes during ontogenesis among individuals of a same species forming a social group may mediate or reduce agonistic interactions, ultimately facilitating the access for breeding positions [49]. The cohabitation of different clownfish species in the same sea anemone host may also support this hypothesis. Indeed, cohabiting clownfishes differ morphologically and always show dissimilar white stripe patterns [38]. While this hypothesis needs to be further explored, interspecific signaling and social selection on visual signals probably operate on the phenotypic divergence of clownfishes.

## Conclusion

Our study highlights a strong link between the diversification of pigmentation pattern in clownfish and the developmental trajectories underlying white stripe formation. This sets up exciting perspectives for the study of color pattern diversity in coral reef fishes, for which an Evo/Devo approach was clearly underused until now. As suggested by our integrated study of clownfishes, the diversification of color patterns results from interplay among developmental, ecological, and behavioral processes.

## Methods

### Coding of striped patterns and associated polymorphisms

Clownfishes were classified into four categories according to their striped pattern at the adult stage [52]: species without vertical stripe (group A) or species having one white vertical stripe on the head (group B) or two (head and trunk) (group C), or three (head, trunk, and caudal peduncle) (group D) (Additional file 1: Table S1). Four species (*A*. *akallopisos*, *A*. *sandaracinos*, *A*. *perideraion*, and *A*. *pacificus*) show an atypical pattern with a white line browsing the dorsal region [53]. This horizontal stripe was not taken into consideration in our study as it may come from a different developmental process. Three species (*A*. *clarkii*, *A*. *melanopus*, and *A*. *polymnus*) are polymorphic with two different patterns observed in natural populations (Additional file 1: Table S1). Accordingly, we repeated all comparative analyses using every combination of coding (i.e., eight combinations).

### Phylogeny, ancestral state reconstruction, and stochastic mapping

We used stochastic character mapping [54] to infer possible histories of the stripe pattern and stripe pattern ontogenesis. The stochastic mapping and the ancestral state reconstruction was produced using the function *make.simmap* in the package phytools (version 0.5.38; [55]) for R [56]. We then sampled 10,000 character histories allowing the incorporation of the uncertainty associated with the timing of the transitions between morphological states. For the parameterization of *make.simmap*, we used the estimated ancestral state and the best model for the transition matrix from our empirical data. To assess the best model for the transition matrix, we fitted a model with equal rate of transition between states and a model with all rates different using the function *ace* in the R-package ape [57]. The likelihood of these two models was then compared using a likelihood ratio test, which suggested the use of unequal rates (see MuSSE results). Statistics for stripe morph histories were retrieved using the function *describe.simmap* from the phytools R-package.

### Model of striped pattern evolution

To test whether the evolution of striped patterns is not random in clownfishes, we compared transition rates between stripe morphs using the “multiple state speciation extinction” (MuSSE) method. MuSSE is an extension of the BiSSE maximum likelihood-based test, which is described in [28, 29]. To test our hypotheses, we used the R-package diversitree [29] to compare the fit of four different models: (i) a model in which all transition rates vary independently, (ii) a model with an equal rate transition matrix (e.g., *q*_*14*_ = *q*_*41*_), (iii) a model in which the loss or the acquisition of two stripes by evolutionary color shift is not allowed (i.e., *q*_*31*_ = *q*_*41*_ = *q*_*42*_ = *q*_*13*_ = *q*_*14*_ = *q*_*24*_ = 0), and (iv) a model combining the constrains of model (ii) and model (iii). In order to reduce the number of parameters, every model assumed that the speciation rates (λ) and the extinction rates (μ) are equal among stripe morphs. Moreover, we corrected for incompletely resolved phylogeny without specifying the state of missing species and we assumed that the missing species are randomly distributed on the phylogenetic tree. The fit of models was compared using sample-size-corrected Akaike’s Information Criterion (AICc) scores and weights [58]. A ΔAICc value of 4 or more was taken as an indication of support for one model over the others [58].

### Ecological and morphological data

Our morphological analysis includes body elongation, body size, and dorsal fin morphologies. Maximum body size was retrieved from Fishbase [59]. Body elongation and dorsal fin morphology quantifications were determined using pictures of *Amphiprion* sp. individuals found in Fishbase [59] or nicely provided by J.E. Randall (http://pbs.bishopmuseum.org/images/JER/) and J. Williams. Others were studied in the marine vertebrate collections of S. Planes (CRIOBE) and Museum of Natural National History (MNHN). This analysis includes 22 species over the 30 described species (Additional file 1: Table S1). The species *A*. *chagosensis*, *A*. *latezonatus*, *A*. *leucokranos*, *A*. *mccullochi*, *A*. *pacificus*, *and A. sebae* are not studied here because of the lack of individuals for quantifications. To quantify body elongation, we used the ratio between body height and the standard length. In order to describe the ingression between the anterior and the posterior lobe of the dorsal fin, we calculated two indexes based on the length of the third dorsal spine (l1), the length of the most posterior spine (l2), the length of the longest soft ray (lr), and the length of the dorsal fin (L). Anterior and posterior lobe morphology indexes correspond to (l1-l2)/L and (lr-l2)/L, respectively (Fig. 6e). Ecological data (i.e., number of host sea anemones) were retrieved from FishBase [59] and the primary literature [33, 60].

### Relationships between striped patterns and fish morphology

Body size and body elongation describe the overall fish shape, and it has been shown to be linked with swimming performance and with adaption towards different macro-habitats [35, 36]. Dorsal fins are associated with stability and thrust [61], and their shape may thus provide information on the swimming behavior of clownfishes. We predicted that the striped pattern of clownfishes is related to ecological and behavioral differences, and thus, phylogenetic generalized least-squares (PGLS) regressions using a Brownian motion model were used to test for correlated evolutionary relationships between the number of stripes and the forms of fish body and dorsal fin. PGLS analyses were performed in the R-package geiger (version 2.0.6; [62]).

### Species recognition hypothesis

The composition of clownfish communities at eight locations was retrieved from Camp et al. [38], and we counted the number of identical striped pairs within each of the eight communities. To test if color similarity among sympatric species was less than would be expected by chance, we generated 9999 random communities by creating random communities using the function *randomizeMatrix* [63] from the R-package *picante* (version 1.6.2; [63]). Variation of species morph pool due to color polymorphism was also included in the generated communities. Then, we compared the number of identical species in each random community to the number in the natural community by a binomial test.

### Larval rearing and observation of color ontogenesis

*A*. *ocellaris* and *A*. *frenatus* were maintained at 26 °C in separate 60-L aquaria. Breeding pairs laid egg clutches on the underside of a terracotta pot placed in their aquarium. On the night of hatching (9 days post laying, 26 °C), egg clutches were transferred from the parental aquarium to a 30-L larval rearing aquarium. Larvae were fed rotifers (*Brachionus plicatilis*) at 10 individuals per milliliter three times a day for the first 7 days. The ratio of *Artemia nauplii* to rotifers was increased each day until larvae were fed only five individuals of *Artemia nauplii* per milliliter from day 7. From day 7 until day 20, larvae are euthanized or anesthetized in MS222 at 200 mg/L and 100 mg/L, respectively, in filtered aquarium water and photographed under a stereomicroscope. At least three larvae per species per day of development are studied.

### Drug treatment of larvae

TAE684 (NVP-TAE684) (HY-10192, MedChem Express), a specific inhibitor of Ltk and Alk [31], was diluted in dimethylsulphoxyde (DMSO; Sigma-Aldrich Louis, MI, USA) to a final concentration of 6 mM. Larvae were treated from 5 until 18 dph in 0.005% DMSO with 0.3 μM or 0.6 μM TAE684 or without (controls). For each condition, five larvae were treated in 500-mL fish medium in a beaker (in total 20 individuals per conditions). One hundred milliliters of solution was changed every day.

## Additional files

Additional file 1: **Table S1.** Dataset list of studied species. Dataset list of studied species, with relative coding based on their color pattern [52], color pattern ontogenesis [64–72], and number of host sea anemones [33] and with quantifications of dorsal fin shape and body elongation. Photographs of specimens for quantifications of dorsal fin shape and body elongation were obtained from online databases (FishBase and “J. E. Randall’s Fish Photos” http://pbs.bishopmuseum.org/images/JER/), or nicely provided by J. Williams. Others were studied in the marine vertebrate collections of S. Planes (CRIOBE) and the Museum of Natural National History (MNHN). Excel document 19 ko. (XLSX 18 kb)

Additional file 2: **Figure S1.** Successive caudo-rostral loss of stripes during evolution is independent of clownfish color polymorphism. Phylogenetic trees of clownfishes from Litsios et al. (2014) [26] with a summary map of white stripe number histories generated through stochastic character mapping. Here, all the combinations of species color pattern polymorphism have been taken into account. Interestingly, all these traits mapping show that the diversification of white striped pattern is a history of loss from an ancestral clownfish having three stripes and that these losses occurred in a progressive fashion in a caudal to rostral sequence. Circles at the tips of the tree indicate each species striped pattern and circles at every internal nodes give probabilities of ancestral striped pattern. PDF document 287 ko. (PDF 286 kb)

Additional file 3: **Table S2.** Summary statistics of the stochastic mapping. Summary statistics of the stochastic mapping (10,000 simulations), assuming that all transition rates (q) among stripe morphs are free to vary. Morphs are coded as followed: species without vertical stripe (A), species having one white vertical stripe on the head (B), species having two white vertical stripes (head and trunk) (C), species having three white vertical stripes (head, trunk, and caudal peduncle) (D). Results are provided for every combination of coding. Word document 13 ko. (DOCX 12 kb)

Additional file 4: **Table S3.** Model fitting of the four striped pattern evolutionary models. Model fitting of the four striped pattern evolutionary models, using the different coding of color patterns detailed in Additional file 1: Table S1. Models are ranked from best to worst, according to AIC scores and Akaike weights (wtAIC). ∆AIC scores indicate the difference between the candidate model and the best-fitting model. According to [58], a ΔAICc value of 4 or more was taken as an indication of support for one model over the other following. Matrices of transition rates (q) among stripe morphs are described for the four evolutionary models. Speciation and extinction rates are assumed to be equal among morphs. See text for details about models and Additional file 1: Table S1 for color coding. Word document 14 ko. (DOCX 13 kb)

Additional file 5: **Table S4.** Relationship between the number of vertical white stripes and eco-morphological variables. Results from PGLS analyses. X and Y refer to the variables in the linear regression model. See Additional file 1: Table S1 for color coding. Word document 14 ko. (DOCX 13 kb)

Additional file 6: **Figure S2.** Lobe morphology index of dorsal fin of clownfish species. Graphs representing the quantifications of posterior (a-b) and anterior (c-d) lobe morphology index in each species. In each graph, we presented the mean from the smallest to the biggest for each stripe morph. (a-c) Graphs represent the mean of posterior (a) and anterior (b) lobe morphology index (dots). Error bars indicate the standard deviation. (b-d) Boxplots of the mean of posterior (c) and anterior (d) lobe morphology index. Boxes extend from the 25th to 75th percentiles, whereas whiskers go down to the smallest value and up to the largest. Median data are indicated by horizontal line within each box. PDF document 67 ko. (PDF 66 kb)
