# Supplementary figures and images for: Ontogenetic and phylogenetic simplification during white stripe evolution in clownfishes
Source: BMC Biol. 2018 Sep 5;16:90. doi: 10.1186/s12915-018-0559-7 (PMC6123960; doi:10.1186/s12915-018-0559-7)

## Coding 1

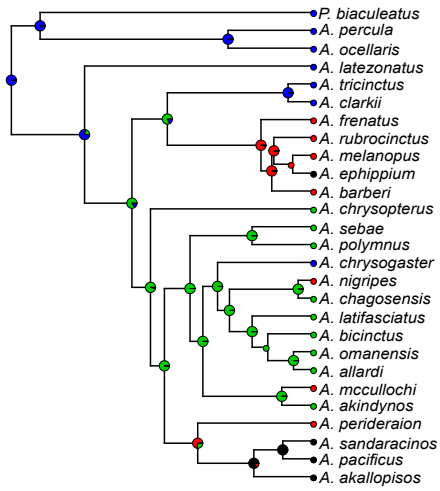

## Coding 2

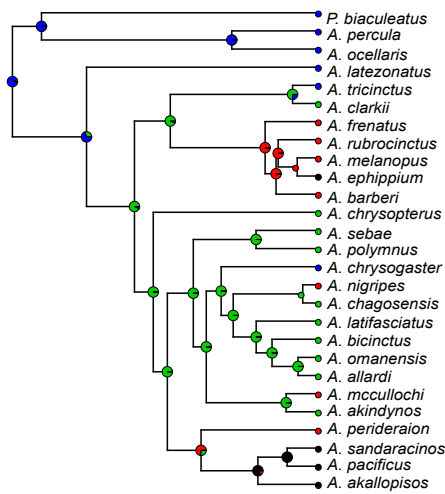

### Coding 3

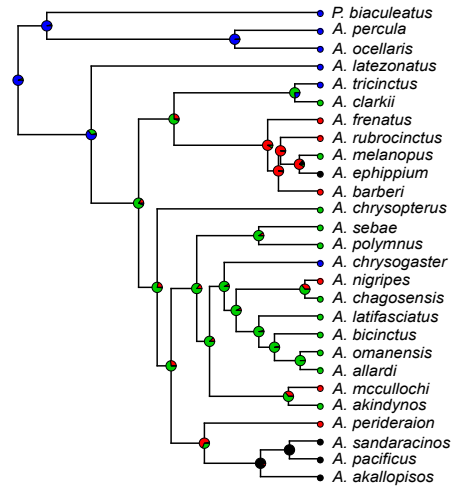

## Coding 4

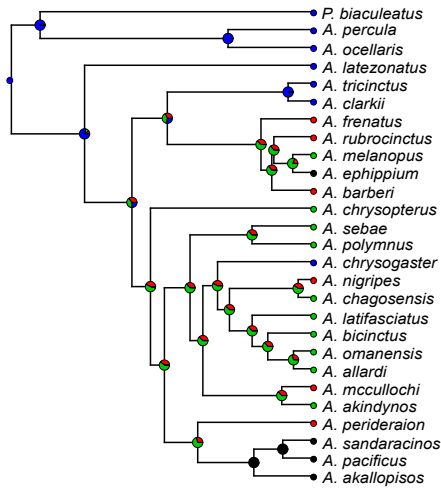

## Coding 5

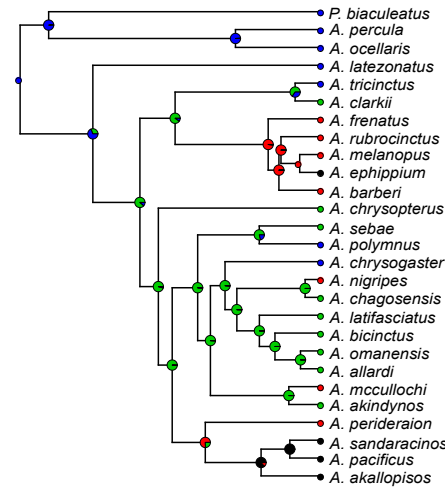

## Coding 6

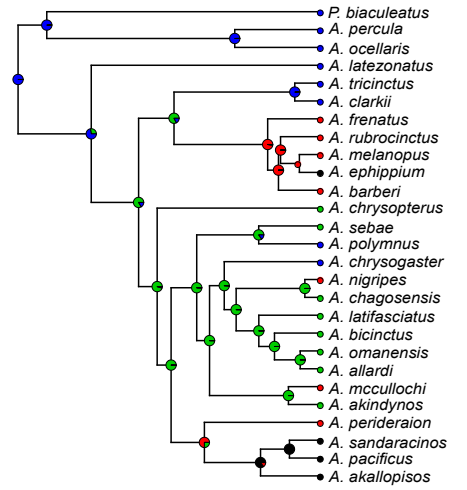

## Coding 7

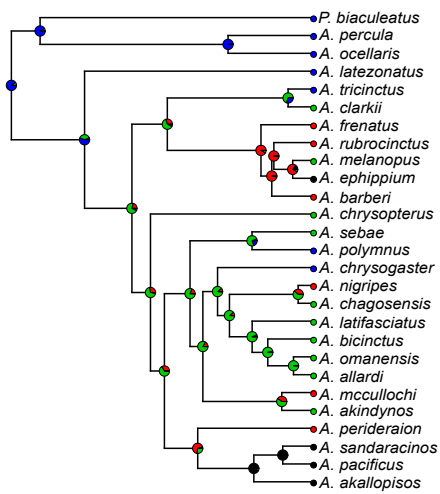

## Coding 8

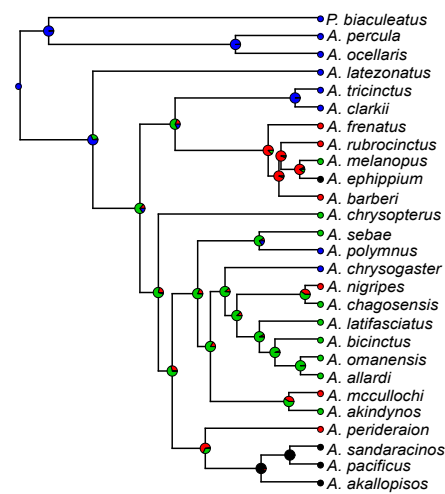

Supplement: Supplementary file 2 — Figure S1. Successive caudo-rostral loss of stripes during evolution is independent of clownfish color polymorphism. Phylogenetic trees of clownfishes from Litsios et al. (2014) [26] with a summary map of white stripe number histories generated through stochastic character mapping. Here, all the combinations of species color pattern polymorphism have been taken into account. Interestingly, all these traits mapping show that the diversification of white striped pattern is a history of loss from an ancestral clownfish having three stripes and that these losses occurred in a progressive fashion in a caudal to rostral sequence. Circles at the tips of the tree indicate each species striped pattern and circles at every internal nodes give probabilities of ancestral striped pattern. PDF document 287 ko. (PDF 286 kb) [file 12915_2018_559_MOESM2_ESM.pdf]

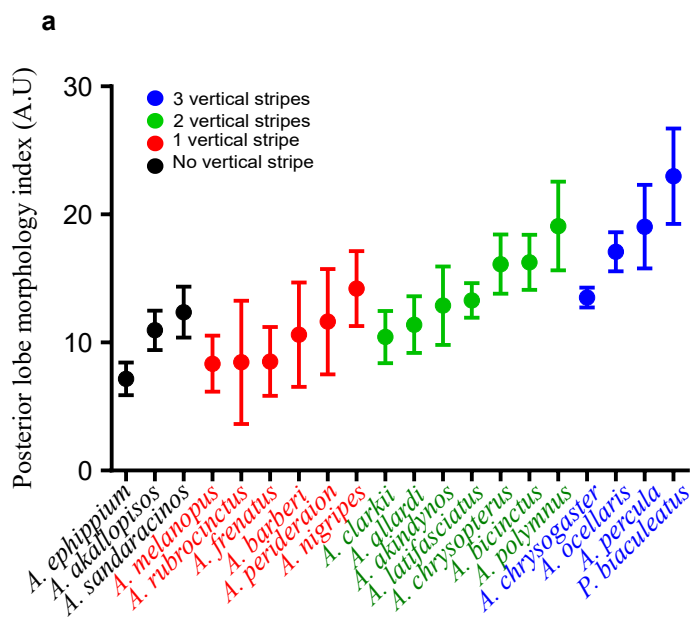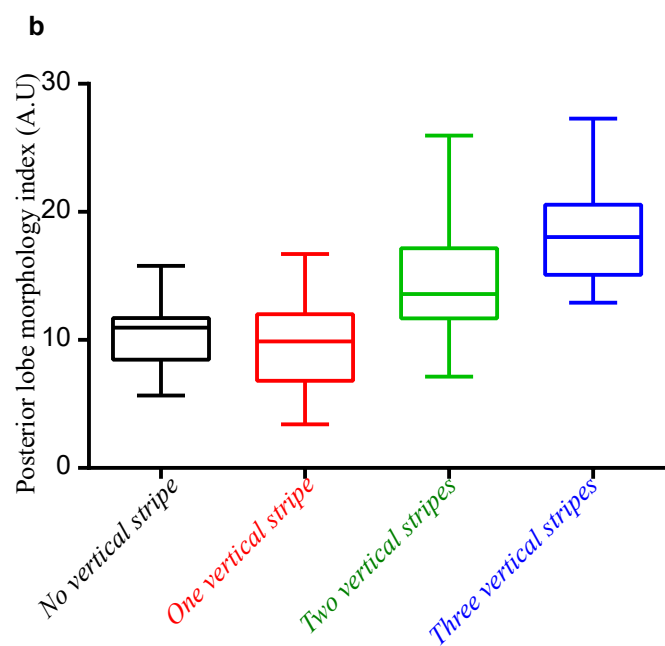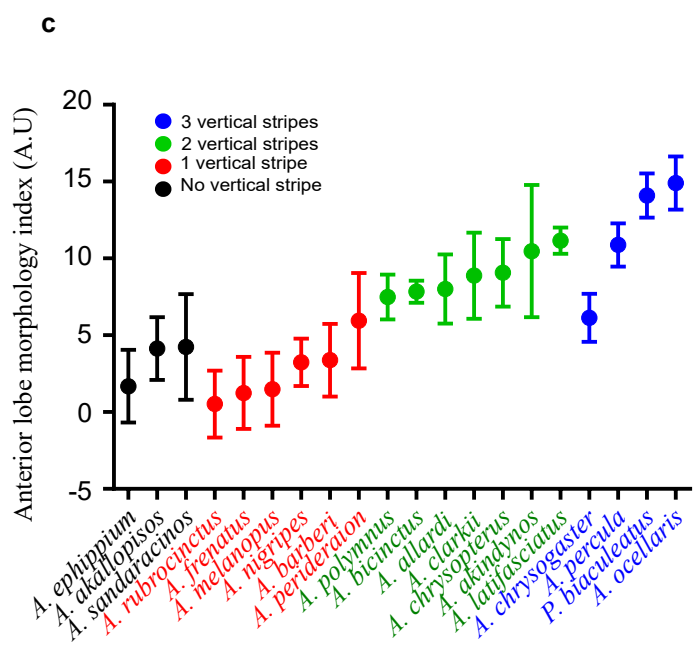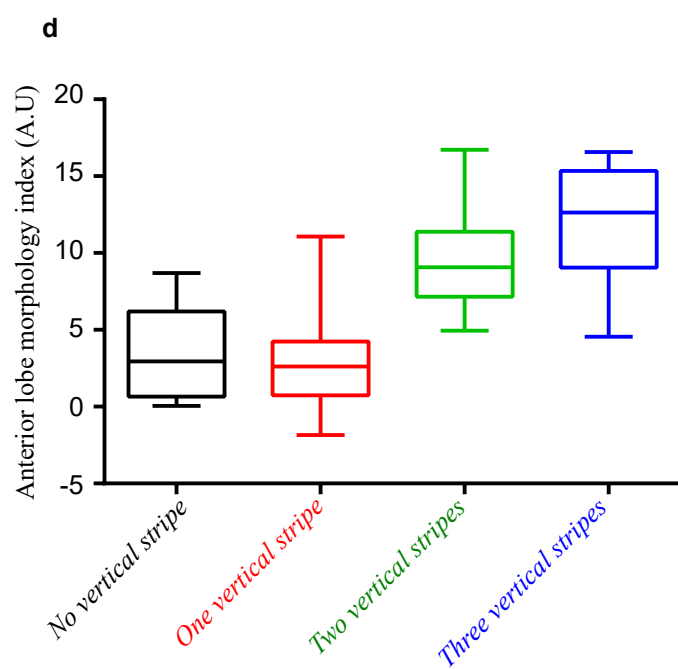

Supplement: Supplementary file 6 — Figure S2. Lobe morphology index of dorsal fin of clownfish species. Graphs representing the quantifications of posterior (a-b) and anterior (c-d) lobe morphology index in each species. In each graph, we presented the mean from the smallest to the biggest for each stripe morph. (a-c) Graphs represent the mean of posterior (a) and anterior (b) lobe morphology index (dots). Error bars indicate the standard deviation. (b-d) Boxplots of the mean of posterior (c) and anterior (d) lobe morphology index. Boxes extend from the 25th to 75th percentiles, whereas whiskers go down to the smallest value and up to the largest. Median data are indicated by horizontal line within each box. PDF document 67 ko. (PDF 66 kb) [file 12915_2018_559_MOESM6_ESM.pdf]
