# Supplementary material for: Ontogenetic and phylogenetic simplification during white stripe evolution in clownfishes
Source: BMC Biol. 2018 Sep 5;16:90. doi: 10.1186/s12915-018-0559-7 (PMC6123960; doi:10.1186/s12915-018-0559-7)
Supplement: Supplementary file 3 — Table S2. Summary statistics of the stochastic mapping. Summary statistics of the stochastic mapping (10,000 simulations), assuming that all transition rates (q) among stripe morphs are free to vary. Morphs are coded as followed: species without vertical stripe (A), species having one white vertical stripe on the head (B), species having two white vertical stripes (head and trunk) (C), species having three white vertical stripes (head, trunk, and caudal peduncle) (D). Results are provided for every combination of coding. Word document 13 ko. (DOCX 12 kb) [file 12915_2018_559_MOESM3_ESM.docx]

Additional file 3

| Transition | Coding 1 | Coding 2 | Coding 3 | Coding 4 | Coding 5 | Coding 6 | Coding 7 | Coding 8 |
| --- | --- | --- | --- | --- | --- | --- | --- | --- |
| q_A,B_ | 0 | 0 | 0 | 0 | 0 | 0 | 0 | 0 |
| q_A,C_ | 0 | 0 | 0.289 | 0 | 0 | 0 | 0.3491 | 0.176 |
| q_A,D_ | 0 | 0 | 0 | 0 | 0 | 0 | 0 | 0 |
| q_B,A_ | 2.158 | 2.165 | 2.190 | 0 | 2.152 | 2.164 | 2.211 | 2.142 |
| q_B,C_ | 0 | 0 | 5.692 | 37.44 | 0 | 0 | 6.255 | 6.575 |
| q_B,D_ | 0 | 0 | 0 | 2.301 | 0 | 0 | 0 | 0 |
| q_C,A_ | 0 | 0 | 0 | 2.037 | 0 | 0 | 0 | 0 |
| q_C,B_ | 4.28 | 4.301 | 7.225 | 37.13 | 4.271 | 4.283 | 7.269 | 7.896 |
| q_C,D_ | 2.85 | 2.424 | 2.891 | 0 | 4.231 | 3.779 | 4.564 | 3.839 |
| q_D,A_ | 0 | 0 | 0 | 0 | 0 | 0 | 0 | 0 |
| q_D,B_ | 0 | 0 | 0 | 1.656 | 0 | 0 | 0 | 0 |
| q_D,C_ | 1.769 | 1.536 | 1.718 | 0 | 2.759 | 2.04 | 2.494 | 2.044 |
| SUM | 11.06 | 10.43 | 20.01 | 80.57 | 13.41 | 12.27 | 23.14 | 22.67 |
