# Supplementary material for: Ontogenetic and phylogenetic simplification during white stripe evolution in clownfishes
Source: BMC Biol. 2018 Sep 5;16:90. doi: 10.1186/s12915-018-0559-7 (PMC6123960; doi:10.1186/s12915-018-0559-7)
Supplement: Supplementary file 4 — Table S3. Model fitting of the four striped pattern evolutionary models. Model fitting of the four striped pattern evolutionary models, using the different coding of color patterns detailed in Additional file 1: Table S1. Models are ranked from best to worst, according to AIC scores and Akaike weights (wtAIC). ∆AIC scores indicate the difference between the candidate model and the best-fitting model. According to [58], a ΔAICc value of 4 or more was taken as an indication of support for one model over the other following. Matrices of transition rates (q) among stripe morphs are described for the four evolutionary models. Speciation and extinction rates are assumed to be equal among morphs. See text for details about models and Additional file 1: Table S1 for color coding. Word document 14 ko. (DOCX 13 kb) [file 12915_2018_559_MOESM4_ESM.docx]

Additional file 4

|  |  | AIC | ∆AIC | wtAIC |
| --- | --- | --- | --- | --- |
| Coding 1 | Model iv | 191.74 | 0.00 | 0.88 |
|  | Model iii | 196.58 | 4.84 | 0.08 |
|  | Model ii | 197.74 | 6.00 | 0.04 |
|  | Model i | 205.58 | 16.84 | 0.00 |
| Coding 2 | Model iv | 188.34 | 0.00 | 0.87 |
|  | Model iii | 192.90 | 4.56 | 0.09 |
|  | Model ii | 194.34 | 6.00 | 0.04 |
|  | Model i | 204.80 | 16.46 | 0.00 |
| Coding 3 | Model iv | 196.11 | 0.00 | 0.89 |
|  | Model iii | 201.15 | 5.05 | 0.07 |
|  | Model ii | 202.11 | 6.00 | 0.04 |
|  | Model i | 213.13 | 17.02 | 0.00 |
| Coding 4 | Model iv | 192.47 | 0.00 | 0.89 |
|  | Model iii | 197.52 | 5.06 | 0.07 |
|  | Model ii | 198.47 | 6.00 | 0.04 |
|  | Model i | 208.97 | 16.50 | 0.00 |
| Coding 5 | Model iv | 194.75 | 0.00 | 0.88 |
|  | Model iii | 199.54 | 4.80 | 0.08 |
|  | Model ii | 200.75 | 6.00 | 0.04 |
|  | Model i | 211.54 | 16.80 | 0.00 |
| Coding 6 | Model iv | 191.73 | 0.00 | 0.85 |
|  | Model iii | 195.93 | 4.19 | 0.10 |
|  | Model ii | 197.73 | 6.00 | 0.04 |
|  | Model i | 207.34 | 15.60 | 0.00 |
| Coding 7 | Model iv | 199.06 | 0.00 | 0.86 |
|  | Model iii | 203.32 | 4.26 | 0.10 |
|  | Model ii | 205.06 | 6.00 | 0.04 |
|  | Model i | 215.30 | 16.24 | 0.00 |
| Coding 8 | Model iv | 201.80 | 0.00 | 0.71 |
|  | Model iii | 204.00 | 2.20 | 0.24 |
|  | Model ii | 207.04 | 5.24 | 0.05 |
|  | Model i | 216.00 | 14.20 | 0.00 |
