# Supplementary material for: Ontogenetic and phylogenetic simplification during white stripe evolution in clownfishes
Source: BMC Biol. 2018 Sep 5;16:90. doi: 10.1186/s12915-018-0559-7 (PMC6123960; doi:10.1186/s12915-018-0559-7)
Supplement: Supplementary file 5 — Table S4. Relationship between the number of vertical white stripes and eco-morphological variables. Results from PGLS analyses. X and Y refer to the variables in the linear regression model. See Additional file 1: Table S1 for color coding. Word document 14 ko. (DOCX 13 kb) [file 12915_2018_559_MOESM5_ESM.docx]

Additional file 5

|  | X | Y | *F-value* | *P-value* |
| --- | --- | --- | --- | --- |
| Coding 1 | Number of vertical stripes | Number of host sea anemones | 0.134 | 0.718 |
|  |  | Body size | 0.027 | 0.871 |
|  |  | Body elongation | 0.638 | 0.434 |
|  |  | Anterior lobe of dorsal fin | **14.530** | **9x10^-04^** |
|  |  | Posterior lobe of dorsal fin | **4.398** | **0.047** |
| Coding 2 | Number of vertical stripes | Number of host sea anemones | 2.779 | 0.109 |
|  |  | Body size | 0.233 | 0.634 |
|  |  | Body elongation | 0.057 | 0.815 |
|  |  | Anterior lobe of dorsal fin | **14.075** | **0.001** |
|  |  | Posterior lobe of dorsal fin | 2.539 | 0.125 |
| Coding 3 | Number of vertical stripes | Number of host sea anemones | 0.282 | 0.601 |
|  |  | Body size | 0.048 | 0.828 |
|  |  | Body elongation | 0.005 | 0.942 |
|  |  | Anterior lobe of dorsal fin | **10.340** | **3x10^-03^** |
|  |  | Posterior lobe of dorsal fin | 2.459 | 0.131 |
| Coding 4 | Number of vertical stripes | Number of host sea anemones | 2.671 | 0.116 |
|  |  | Body size | 0.239 | 0.629 |
|  |  | Body elongation | 0.916 | 0.351 |
|  |  | Anterior lobe of dorsal fin | **9.830** | **4.6x10^-03^** |
|  |  | Posterior lobe of dorsal fin | 1.350 | 0.258 |
| Coding 5 | Number of vertical stripes | Number of host sea anemones | 0.112 | 0.741 |
|  |  | Body size | 0.012 | 0.915 |
|  |  | Body elongation | 0.440 | 0.515 |
|  |  | Anterior lobe of dorsal fin | **13.083** | **1.4x10^-03^** |
|  |  | Posterior lobe of dorsal fin | **6.220** | **0.020** |
| Coding 6 | Number of vertical stripes | Number of host sea anemones | 2.495 | 0.128 |
|  |  | Body size | 0.170 | 0.684 |
|  |  | Body elongation | 0.106 | 0.748 |
|  |  | Anterior lobe of dorsal fin | **12.627** | **1.7x10^-03^** |
|  |  | Posterior lobe of dorsal fin | 3.893 | 0.606 |
| Coding 7 | Number of vertical stripes | Number of host sea anemones | 0.252 | 0.620 |
|  |  | Body size | 0.029 | 0.866 |
|  |  | Body elongation | 0.023 | 0.882 |
|  |  | Anterior lobe of dorsal fin | **9.680** | **4.9x10^-03^** |
|  |  | Posterior lobe of dorsal fin | 3.559 | 0.072 |
| Coding 8 | Number of vertical stripes | Number of host sea anemones | 2.466 | 0.130 |
|  |  | Body size | 0.186 | 0.670 |
|  |  | Body elongation | 1.026 | 0.324 |
|  |  | Anterior lobe of dorsal fin | **9.197** | **5.9x10^-03^** |
|  |  | Posterior lobe of dorsal fin | 2.171 | 0.154 |
